# Threshold intensity factors as lower boundaries for crack propagation in ceramics

**DOI:** 10.1186/1475-925X-3-41

**Published:** 2004-11-17

**Authors:** Rudolf Marx, Franz Jungwirth, Per-Ole Walter

**Affiliations:** 1 Department of Prosthetic Dentistry, Section of Dental Materials, University Hospital of the University of Technology, 52074 Aachen, Germany

## Abstract

**Background:**

Slow crack growth can be described in a v (crack velocity) versus K_I _(stress intensity factor) diagram. Slow crack growth in ceramics is attributed to corrosion assisted stress at the crack tip or at any pre-existing defect in the ceramic. The combined effect of high stresses at the crack tip and the presence of water or body fluid molecules (reducing surface energy at the crack tip) induces crack propagation, which eventually may result in fatigue. The presence of a threshold in the stress intensity factor, below which no crack propagation occurs, has been the subject of important research in the last years. The higher this threshold, the higher the reliability of the ceramic, and consequently the longer its lifetime.

**Methods:**

We utilize the Irwin K-field displacement relation to deduce crack tip stress intensity factors from the near crack tip profile. Cracks are initiated by indentation impressions. The threshold stress intensity factor is determined as the time limit of the tip stress intensity when the residual stresses have (nearly) disappeared.

**Results:**

We determined the threshold stress intensity factors for most of the all ceramic materials presently important for dental restorations in Europe. Of special significance is the finding that alumina ceramic has a threshold limit nearly identical with that of zirconia.

**Conclusion:**

The intention of the present paper is to stress the point that the threshold stress intensity factor represents a more intrinsic property for a given ceramic material than the widely used toughness (bend strength or fracture toughness), which refers only to *fast *crack growth. Considering two ceramics with identical threshold limits, although with different critical stress intensity limits, means that both ceramics have identical starting points for *slow *crack growth. Fast catastrophic crack growth leading to spontaneous fatigue, however, is different. This growth starts later in those ceramic materials that have larger critical stress intensity factors.

## Background

Slow crack growth is most suitably described in a *v *(crack velocity) versus *K*_*I *_(stress intensity factor) diagram. Slow crack growth in ceramics is attributed to corrosion assisted stress at crack tips or at any defect pre-existing in the ceramic [1]. The combined presence of body fluid molecules (mainly water), which reduce the surface energy at the crack tip, and the presence of high stresses are the reasons for subcritical crack growth (SCCG) in ceramics.

The presence of stress intensities above a critical value (K_I _> K_Ic_) initiates fast catastrophic crack growth, followed by the deterioration of a dental or a body restoration machined from ceramics. The presence of stress intensities above a threshold value (K_I _> K_I0_) initiates SCCG in ceramics, followed by a slow, however continuous, erosion of the strength of a restoration which also may result in final fatigue. In an early stage of ceramic research it was believed that this lower limit for SCCG is very close to zero. In the mean time, however, one has learned that for most ceramic materials the lower limit for SCCG is significantly larger than zero. Indeed, it may even be just below *K*_*Ic*_.

The threshold limit *K*_*I0 *_corresponds to a crack equilibrium at null crack velocity. Therefore, it allows a safety range of clinical use. The higher the value of *K*_*I0*_, the higher the reliability, and hence the lifetime of a restoration. Bio-components should be designed to work in a region of the *v-K*_*I*_*-*diagram where the upper border line of that region corresponds to the threshold limit.

In the present paper we preferentially focus on those ceramics that are important in dental research. Note, however, that alumina and zirconia have meaning in both fields of application (dentistry and medicine). We use soda lime glass as a well characterized standard and silicon nitride as important in the general field of ceramics.

There are several methods available and in the literature extensively described how the threshold limit can be measured. The feasibility of these measurement procedures is mostly demonstrated with the help of soda lime glass as a brittle solid model.

In principle, the proper test for existence of a threshold lies in the observation of reversibility of crack growth. The threshold can be regarded as a Griffith quiescent point, where forward and backward fluctuations just balance, i.e., the mean velocity of the crack tip becomes zero. The forward and backward fluctuations take place over discrete energy barriers definable as *G *= *W *= 2*γ*, where G is the energy release rate, *W *is the Dupré work of adhesion, and *γ *is the surface energy. If *G *<*W *the crack should retract and heal; otherwise it should repropagate [2]. On the basis of this assumption, the authors in [2] (see also [3]) calculate equations prescribing the *v G *characteristics (crack velocity versus mechanical energy release rate; equivalent to *v *- *K*_*I *_crack velocity versus stress intensity factor) at specified chemical concentrations and temperatures, which can describe observed *v-G *dependencies.

One common experimental method to determine the threshold limit is to measure slow crack growth rate down to velocities as low as 10^-14 ^m/s. Then one can extrapolate from the vertical branch of the function to the zero velocity limit on the stress intensity factor axis K_I_, with the intersection K_I _equal to K_I0 _[4-7].

Another method to determine the aforesaid threshold limit is the "interrupted static fatigue test" (ISF-test) [8]. For a bending experiment, the applied stress is chosen such that a significant fraction of samples fails in a "hold period". Samples that do not fail during this static phase are then fractured by the usual four point bending technique. The threshold is calculated either from the applied stress intensity factor at which 50% of samples fail during the stress hold, or by using the factor applied to the weakest specimen during the stress hold as calculated for various hold times. Once the value of the stress intensity factor becomes independent of hold time, it is equivalent to the threshold [9].

Another method uses a side grooved specimen with a crack propagating along its length, and under a bending condition similar to four point bending. The crack velocity can be obtained from the rate of load relaxation at constant displacement and the initial crack length. Having established the *v *- *K *diagram, the threshold is determined as described above. For further details refer to [10].

Other methods may be characterized by the phrase "decay of residual stress" [11]. Here, the threshold limit can be calculated from the residual stress factor attributed to this decay of residual stress.

The current method of measurement used, however, is based on indentation cracking, analogous to other studies also utilizing flaw initiation for starting the test [11-13]. After this start, however, the subsequent procedure is different. A follow up of the decay of residual stress intensities near the crack tip is done over a period of about one year, determining K_tip _via the COD for different times after indentation [14].

## Methods

Using a micro-hardness testing machine, a Vickers indentation is made on the carefully polished surface of a sample of the ceramic to be investigated. Radial cracks emanate from each of the four indentated corner sources.

To determine the stress intensity present at the crack tip due to the indentation, the near crack tip profile is determined using a scanning microscope (ESEM: "Environmental Scanning Electron Microscope"). A specific feature of this technique is that it is carried out at a moderate vacuum (p ≈ 10^-1 ^mbar). Hence, there is no longer need to sputter the samples with a gold or carbon layer. Our initial attempts to measure the crack opening displacement (COD) showed that sputtering resulted in blurring the crack banks or even partly hiding the crack. Thus, we abandoned those attempts and started again when the ESEM was available. Before the availability of the ESEM it was nearly impossible to precisely measure crack profiles at submicrometer resolution which, however, is mandatory.

**Figure 1:**
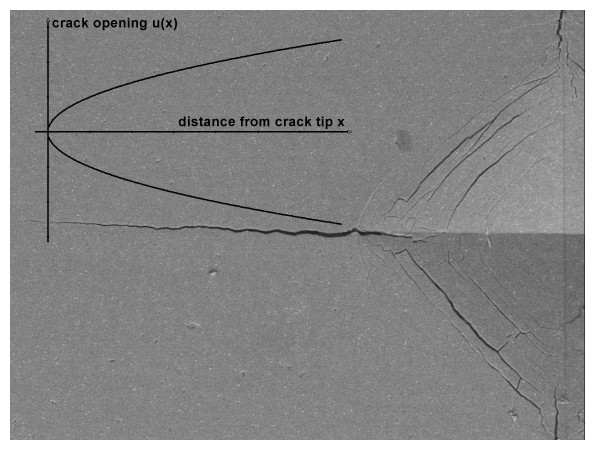
Example of a Vickers indentation. Only one of four corners is shown (length of diagonal 115 *μ*m). With the crack tip as a starting point (*x *= 0) the crack width 2*u(x) is measured at the distance *x *(COD after *Irwin *[15]; ceramic material for this example: Empress 1). The residual tensions cause crack growth over a long time interval until, at the end of the crack, *K*_*tip *_is equal to *K*_*I0*_. Crack tip shielding by secondary effects (micro structural elements which toughen material as the crack extends) may slightly distort results (measured *K*_*I0 *_then lower than true *K*_*I0*_). Insert: idealized COD.

Images of the crack profiles (Fig. 1) were digitally stored and analyzed by imaging software (Paint Shop Pro, V. 6, Jasc Software, Eden Prairie, Maine, USA).

The measured profiles can be attributed to the crack opening displacement (COD) near the crack tip [14]. The near crack tip profiles for stress-free crack surfaces are usually represented by the Irwin K-field displacement relation [15], with 2u being the total COD, *x *the distance from the crack tip, and the plane strain Young's modulus *E' *= *E*/(1-*v*^2^);(*v *= Poisson's constant) being.

We assume that there is no crack shielding. Then, in equilibrium, the currently acting crack tip stress intensity factor *K*_*tip *_is balanced by the toughness of the material *K*_*Ic *_(mode I loading [15]):



and by re-arrangement:



If data are taken sufficiently close to the crack tip (*x *≤ 20 *μ*m), a linear relationship is experimentally observed between *u(x)*^2 ^and *x*. *K*_*tip *_can then be calculated from a regression analysis as the slope of a straight line, provided *E' *is known (see below).

The residual stresses close to the crack tip initiated by the indentation impression gradually decay over time *t*, and one anticipates that they slowly fade away eventually approaching zero. Hence *K*_*tip*_= *K*_*tip*_*(t) *and it is plausible to assume *K*_*tip*_(*t*→∞) ≈ *K*_*I0*_. Therefore, in the present work, because of slow crack growth, we take the threshold value of the stress intensity factor as the time limit of the slowly decreasing *K*_*tip *_value. Provided that a suitable high resolution scanning microscope is at hand, there is no need of sputtering the samples, and the presently utilized method is very simple. A potential shortcoming, however, is that this method may need many months or even years until the residual stresses are relaxed and the threshold value is reached.

The authors concede that they have chosen to consider a somewhat ideal situation since the assumption *K*_*tip*_(*t*→∞) = *K*_*I0 *_assumes ideal behavior. In real ceramics, especially polycrystalline and composite materials, the crack tip may be shielded from residual load by micro structural elements, which toughen the material in the region just before the crack tip [2]. This behavior is reminiscent to R-curve behavior.

We carried out ESEM analyses of crack profiles after 1 hour and then after up to 420 days, at 5 dates distributed over the whole time interval (Fig. 3). After indentation and between two measurements the samples were stored at normal lab environmental conditions (21°C, 65 % humidity).

We determined the threshold stress intensity of the following ceramics (Soda lime glass and veneering ceramics as reference): Al_2_O_3_, coarse grained, load of indention 9,9 kg, Young's modulus 350 GPa (Frialit-Degussit, Mannheim/Ludwigshafen, Germany), Cerec Mark II, 4 kg, 69 GPa, HiCeram, 6,9 kg, 107 GPa, VMK 95, 4 kg, 91 GPa (all three Vita, Bad Säckingen, Germany), Cercon Base, 7,9 kg, 210 GPa, CergoGold, 4 kg, 70 GPa (both Degudent-Dentsply, Hanau, Germany), Dicor, 2 kg, 74 GPa (Corning Glass Works, Corning, USA), Empress 1, 5,9 kg, 67 GPa, Empress 2, 5,9 kg, 96 GPa (both Ivoclar, Schaan, Liechtenstein), Lava, 8 kg, 210 GPa (3M-Espe, Seefeld, Germany), Soda lime glass, 2 kg, 73 GPa (Saint Gobain, Aachen, Germany), Si_3_N_4_, 6 kg, 289 GPa and hipped 5%Y_2_O_3_-Zirkon, 8 kg, 210 GPa.

The constitution of the soda lime glass was SiO_2 _72.65, Al_2_O_3 _0.28, MgO 3.98, CaO 8.84, Na_2_O 13.79, K_2_O 0.19, other 0.27.

## Results

**Figure 2:**
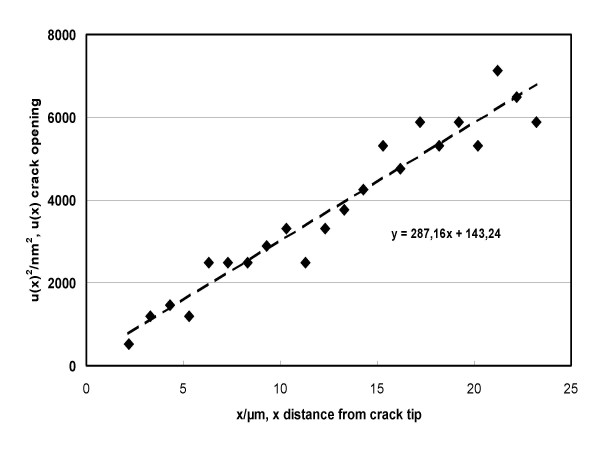
Regression analysis representing data for "Cerec Mark II" two days after indentation, analyzed with the help of Eq. 1 (*u(x)*^2 ^as a function of distance from crack tip *x *(2 *μ*m <*x *< 23 *μ*m)). A linear relationship is observed.

**Figure 3:**
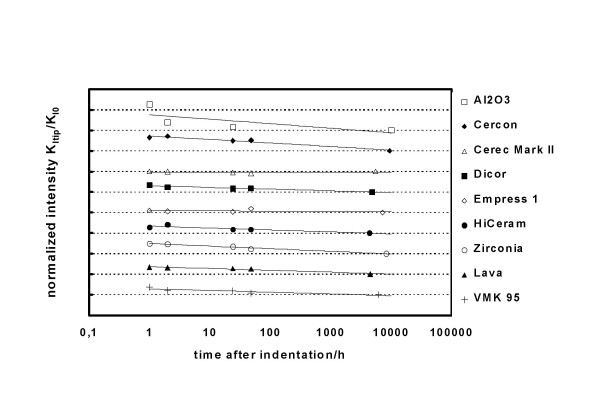
*K*_*tip*_(*t*) values of nine out of the thirteen ceramics investigated. The gradual decrease of *K*_*tip*_(*t*) with time due to decaying stress intensity at the crack tip becomes apparent.

As examples, Fig. 1 shows a crack starting at the corner of a Vickers indentation (right hand) and Fig. 2 shows a plot representing data for "Cerec Mark II" two days after indentation, as a function of distance from crack tip *x *(2 *μ*m <*x *< 23 *μ*m), analyzed with the help of Eq. 1'. A linear relationship is observed, from which the value of *K*_*tip *_(*t *= *48 h*) = *0,90 MPa√m *was easily and precisely deduced. Fig. 3 shows all *K*_*tip *_values determined in an analogous manner for nine examples out of the thirteen investigated ceramics. The gradual decrease of *K*_*tip*_*(t) *due to decaying stress intensities at the crack tip becomes apparent. The manner in which *K*_*tip*_*(t) *decreases suggests an exponential relationship, as the decrease appears to be linear on a logarithmic scale. The truncation of the measurements after about 10^4 ^hours (for reasons of feasibility) appears somewhat arbitrarily, and it cannot be excluded that a further decay, although very small, may have been missed. Note that due to the apparent exponential relationship, the overestimation of the threshold value *K*_*I0 *_due to the truncation after 10^4 ^hours becomes smaller and smaller with time. We plan to do further measurements after another interval of 10^4 ^hours (417 days). Considering the mathematical aspect, 10^5 ^hours (11+ years) would make more sense; but such a long interval is obviously not practicable. As already mentioned, this time constraint is a decided disadvantage of our current method to determine the threshold value.

**Figure 4:**
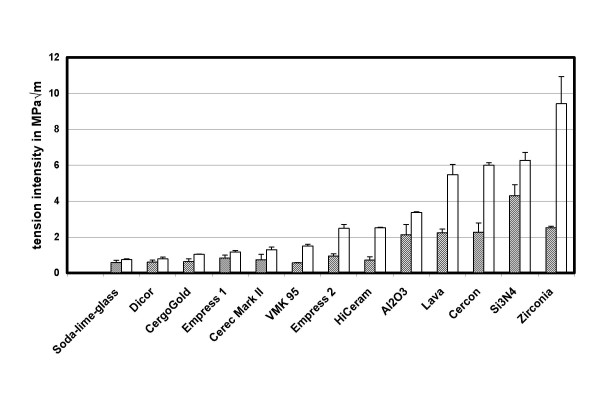
*K*_*I0 *_threshold values (hatched columns) in comparison with their counterpart critical stress intensities, *K*_*Ic *_(unfilled columns). Refer also to [22]. In the available literature, values for reference: Al_2_O_3 _(K_I0 _= 2.5 ± 0.2 MPa√m); ZrO_2 _(K_I0 _= 3.1 ± 0.2 MPa√m, both values after [4]); Soda lime glass (K_I0 _= 0,42 MPa√m, after [11]).

Being aware of the these limitations, and having in mind the neglected possible crack tip shielding as discussed above, we identify *K*_*I0*_= *K*_*tip*_(*t*→∞). Fig. 4 displays all *K*_*I0 *_values in comparison with their *K*_*Ic *_counterparts.

## Discussion

*K*_*Ic *_is the lower limit for (fast) catastrophic crack growth. Stress intensities exceeding this limit cause fast crack growth at supersonic velocity, and eventually result in destruction of ceramic components. This kind of destruction, however, is not the most common or important, since it can be avoided by strictly limiting the stress intensities existing throughout a component by a suitable shape of construction.

**Figure 5:**
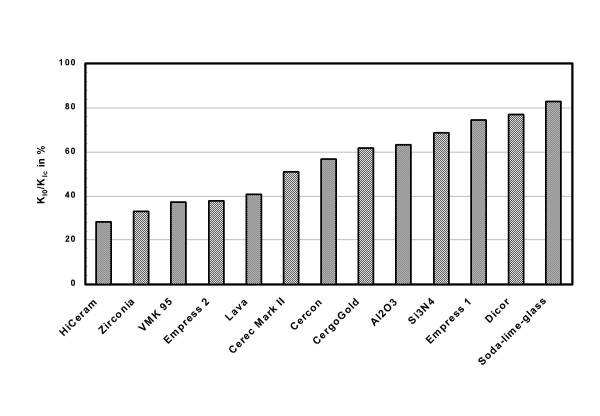
Ranking of all ceramics as imposed by their ratio "threshold value to critical value" (*K*_*I0*_/*K*_*Ic*_). Dicor: see [23].

*K*_*I0 *_is the upper limit of stress intensities for absence of crack growth and the lower limit for (slow) subcritical crack growth (SCCG). Limiting stress intensities such that they stay always below *K*_*I0 *_means infinite life time for a component, since SCCG becomes irrelevant. Hence, the most favorable characteristic stress intensity values are obvious: *K*_*Ic *_as high as possible and *K*_*I0 *_as close as possible to *K*_*Ic*_. Such a selection minimizes the extension of the interval in which subcritical crack growth can take place, and it maximizes resistance to catastrophic crack growth due to overloading. Fig. 5 gives a ranking of all ceramics currently tested, based on threshold values related to the corresponding critical values *K*_*I0*_*/K*_*Ic*_. Favorable ceramics within their class of toughness are situated at the right hand side of the chart. Note, however, that a perfect ceramic material dependent on the focused area of application has not only a favorable (threshold/critical) stress strength relationship but also a high *K*_*Ic *_value.

At first glance zirconia may seem to be a ceramic material superior to alumina, since it has a critical stress intensity factor (Fig. 4: 9.4 ± 1.5 MPa·√m) which is about three times larger than this of alumina. Values in the literature for zirconia are up to about 8 MPa·√m [16], compared with 5.4 MPa·√m and [5]: 5.0 ± 0.2 MPa·√m [17] for alumina. Naturally, this is a significant advantage when operations near the critical stress of a material are involved. However, in practical applications, stresses having an intermediate level are more common, thus initiating SCCG instead of catastrophic crack growth. Then, if the threshold stress intensities of two ceramics are equal, they are both subject to SCCG at the same rate. Apparently. zirconia vs alumina is an example for such a situation (Fig. 4): meaning that both ceramics have equal potential for SCCG. The different behavior of these ceramics is solely rendered to stress bearing capabilities near catastrophic crack growth. At such stresses near *K*_*Ic *_zirconia, of course, has properties superior to alumina.

It becomes apparent that at moderate stresses alumina and zirconia may be equally suitable choices, and other criteria may become important for favoring the one or the other material. Such reasons may be the ease of shaping, questions of color, ease of veneering, esthetic considerations, availability, and other circumstances.

There is one other aspect to be considered when comparing zirconia and alumina. The exponents *n *of SCCG of both ceramics are high (in principle meaning slow SCCG), and the answer to the question of which of the materials has the larger exponent depends on whether static or cyclic behavior is addressed: *n*_static _= 39 vs 104 and *n*_cyclic _= 28 vs 16 for Al_2_O_3 _and Y-PSZ, respectively [16]. These parameters show that lifetimes are shortened and crack growth rates are significantly accelerated by cyclic loading compared to static loading.

Zirconia is known to be sensitive to humidity, which is a particular important issue when prosthetic and orthopedic applications are considered. It is known that yttria stabilized zirconia ceramics can be destabilized during the process of steam sterilization. This is due to hydrothermal transformation, resulting in surface roughening of the zirconia ceramic femoral heads. These femoral heads may also undergo slow degradation during long term implantation in the human body. This low temperature degradation does not become significant before several years, but it does raise the question of the use of zirconia for load bearing systems [4]. In conclusion, it can be stated that SCCG of Y-TZP is activated by the influence of water [18,19], however, there is some controversy about this effect [20]. An analogous statement holds for MgO-partially stabilized zirconia (PSZ) [21].

Note that concerning the sensitivity to humidity, there is a notable difference between ceramics for dental or for orthopedic applications. Ceramics for dental applications are often veneered by a different ceramic, which means that there is a protective shield against humidity attacking from outside of the ceramic tooth (but not from inside or from the marginal region).

**Figure 6:**
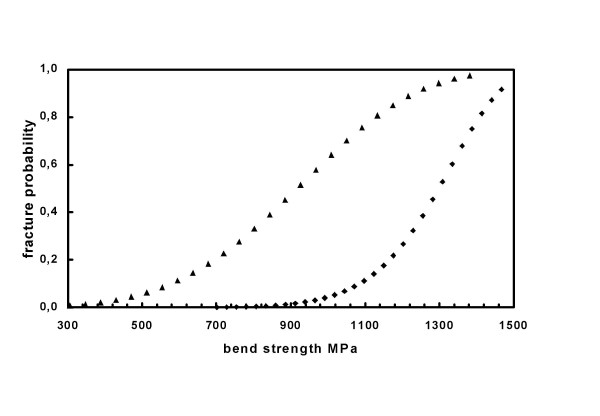
Example (linear Weibull plot) for a zirconia based ceramic material developed for dental applications. Samples handled at 60 % relative humidity (lab environmental conditions; diamonds) vs samples stored in aqua dest for 10 days (triangles). The sensitivity to humidity is obvious. The bending strength due to water storage decreases from *σ*_63% _= 1,346 MPa to 1,003 MPa (about 25 %).

Fig. 6 displays an example of a zirconia ceramic material developed for dental applications and which was formerly used. The sensitivity to humidity becomes apparent.

There are some other examples of ceramics for which a large difference in the critical stress intensities is observed whereas the threshold values are very similar. For these ceramics an analogous argument holds, as given above for alumina vs zirconia. From Fig. 4, for Empress 1 or Empress 2 (e.g.) the following values are measured: K_Ic _= 1.17 ± 0.08 MPa·√m or K_Ic _= 2.48 ± 0.22 MPa·√m, respectively; and K_I0 _= 0.83 ± 0.16 MPa·√m or K_I0 _= 0.94 ± 0.12 MPa·√m, respectively. Again, the critical stress intensity values are largely different, the threshold values, however, are nearly identical. Compare also "Al_2_O_3_" with "Lava" and "Cercon".

## Authors' contributions

RM conceived in the study, designed the study and drafted the manuscript. POW and FJ carried out the experimental work. All authors read and approved the final manuscript. All authors contributed equally to this work.
